# Supplementary material for: Clinical Features and Vaccination Effects among Children with Post-Acute Sequelae of COVID-19 in Taiwan
Source: Vaccines (Basel). 2024 Aug 12;12(8):910. doi: 10.3390/vaccines12080910 (PMC11359259; doi:10.3390/vaccines12080910)
Supplement: Supplementary file 1 [file vaccines-12-00910-s001.zip › vaccines-3052860-supplementary.pdf]

Supplementary Materials

Table S1. Distribution of clinical symptoms during the acute phase among 500 children with PASC, sorted by age.

Symptom/Age	0	1	2	3	4	5	6	7	8	9	10	11	12	13	14	15	16	17
Fever	3.8	4.8	5.0	5.0	7.8	4.6	6.4	5.6	4.8	5.2	5.2	3.8	2.2	3.4	2.2	1.8	2.0	1.6
Cough	3.2	2.4	3.4	3.6	4.4	3.8	4.8	4.0	2.6	4.2	4.2	3.8	1.8	2.6	2.2	2.4	2.2	1.8
Rhinorrhea/nasal congestion	2.6	1.6	3.4	2.8	3.6	2.6	3.6	3.4	2.0	3.4	3.6	2.2	1.8	2.8	2.4	2.4	2.8	1.6
Sore throat	1.6	1.4	2.0	2.2	2.6	2.2	3.0	3.6	2.4	2.4	4.0	2.4	2.6	3.4	2.6	2.6	3.0	1.6
Fatigue	1.0	1.2	1.2	1.2	2.8	1.6	2.4	2.8	2.0	3.2	3.0	2.8	1.8	2.0	1.8	1.2	1.6	1.2
Headache/dizziness	0.0	0.0	0.0	1.0	1.2	1.2	2.2	2.2	2.4	3.0	3.6	3.0	1.2	3.2	2.0	2.4	2.4	1.4
Sneezing	1.6	1.4	1.8	1.0	2.2	1.0	2.4	1.8	0.8	1.2	2.0	1.0	0.8	1.4	1.6	1.0	1.6	0.8
Sputum	0.6	1.0	2.0	0.8	1.4	1.6	2.4	2.2	0.6	1.6	1.0	2.6	0.8	1.6	0.6	1.6	1.6	1.2
Chills	1.2	0.4	0.8	1.2	1.8	1.2	2.2	1.2	1.8	2.2	1.4	2.0	1.2	1.2	0.6	0.4	1.0	0.8
Muscle soreness	0.0	0.0	0.4	0.0	1.0	0.4	1.0	0.6	1.0	1.8	1.6	1.0	1.2	0.8	1.0	0.6	1.0	1.2
Diarrhea	1.0	0.8	0.6	1.4	1.0	0.8	0.8	0.6	0.8	1.0	1.6	0.6	0.6	0.2	0.6	0.4	0.6	0.2
Dyspnea	0.4	0.8	0.0	0.0	0.2	0.0	0.4	0.6	0.6	1.0	1.0	0.8	0.4	0.6	1.6	0.8	1.6	0.2
Abdominal pain	0.0	0.0	0.2	0.8	1.0	1.2	0.4	0.6	0.8	1.6	1.6	0.6	0.4	0.0	0.8	0.6	0.2	0.2
Chest pain	0.0	0.0	0.2	0.2	0.0	0.0	0.6	0.8	0.4	0.8	1.4	0.4	0.4	1.2	1.2	0.6	1.4	0.8
Cold sweating	0.8	0.0	0.0	0.8	1.2	0.8	0.6	0.4	0.8	1.4	0.2	0.8	0.0	0.4	0.4	0.6	0.6	0.2
Nausea	0.0	0.2	0.0	0.6	0.0	0.0	0.2	1.2	0.8	1.6	1.0	0.2	0.0	0.6	0.6	0.6	0.6	0.2
Other symptoms	0.4	0.6	0.6	0.4	0.6	0.2	0.8	0.6	0.4	0.6	0.6	0.2	0.4	0.4	0.4	0.4	0.4	0.4
Joint pain	0.0	0.0	0.0	0.2	0.2	0.4	0.6	0.2	0.2	0.4	0.4	0.6	0.4	0.4	0.0	0.0	0.4	0.2
Seizure	0.0	0.0	0.4	0.2	0.0	0.2	0.2	0.0	0.0	0.2	0.0	0.0	0.0	0.2	0.2	0.2	0.0	0.2
Lost taste	0.0	0.0	0.0	0.0	0.0	0.2	0.2	0.0	0.0	0.0	0.0	0.2	0.0	0.6	0.0	0.2	0.6	0.0
Lost smell	0.0	0.2	0.0	0.0	0.0	0.0	0.2	0.0	0.0	0.0	0.0	0.0	0.0	0.6	0.0	0.2	0.4	0.0
Loss of consciousness	0.0	0.0	0.0	0.0	0.0	0.0	0.0	0.2	0.0	0.0	0.2	0.0	0.0	0.4	0.2	0.0	0.2	0.2
Conjunctival injection	0.0	0.0	0.0	0.2	0.0	0.0	0.0	0.2	0.2	0.0	0.0	0.0	0.0	0.0	0.0	0.2	0.2	0.0
Hemoptysis	0.0	0.0	0.0	0.0	0.0	0.0	0.0	0.0	0.0	0.0	0.0	0.0	0.2	0.0	0.0	0.2	0.2	0.0

Table S2. Distribution of clinical symptoms during the post-COVID-19 phase among 500 children with PASC, sorted by age.

Clinical Symptoms/Age	0	1	2	3	4	5	6	7	8	9	10	11	12	13	14	15	16	17
Cough	2.4	2.4	3.4	2.6	5.4	3.4	4.5	2.8	2.8	2.4	2.6	1.7	0.9	1.1	1.1	1.9	0.9	1.1
Fatigue	1.9	1.5	2.1	2.1	3.4	1.7	2.3	3.2	2.5	2.5	2.3	2.3	1.3	2.1	2.5	2.3	2.9	1.3
Throat problem	1.7	2.2	2.2	1.9	3.7	1.9	1.9	1.9	1.9	1.9	1.5	1.7	1.1	1.1	1.1	1.7	1.5	0.9
Short of breath	1.3	1.5	1.9	1.3	1.0	1.5	1.7	2.9	1.5	2.1	2.9	1.9	0.6	2.3	2.1	1.5	2.3	0.6
Limit daily activity	0.6	0.6	0.9	1.7	1.7	0.9	2.4	2.8	1.7	2.6	2.2	2.4	1.3	1.7	2.8	1.3	2.2	1.3
Body weight change	1.1	1.7	1.7	1.9	1.5	2.4	2.2	1.3	1.7	2.2	2.6	2.2	1.1	1.7	1.9	0.4	2.2	1.1
Attention disturbance	0.6	0.6	1.3	2.8	1.7	1.3	1.3	2.2	2.2	2.8	1.5	1.1	0.6	1.5	1.7	1.5	2.2	1.1
Lack of motivation	0.6	0.4	0.4	1.9	1.7	0.2	2.2	1.5	1.1	1.7	2.4	1.9	1.1	1.9	2.6	2.2	2.4	1.1
Chest pain	0.0	0.4	0.2	0.6	0.4	0.9	2.4	2.6	1.9	1.7	2.2	2.2	0.9	1.9	2.4	1.7	2.2	1.5
Decreased appetite	1.5	1.5	1.1	1.9	1.5	1.3	1.3	1.5	1.3	1.9	1.1	0.9	1.1	1.1	2.2	1.1	1.9	0.4
Anxiety	0.4	0.4	0.9	1.3	1.1	0.9	1.5	1.7	0.9	2.2	1.9	1.3	1.5	1.7	2.6	1.3	1.7	1.3
Sleep disturbance	1.3	2.2	2.2	1.3	2.6	0.9	1.3	1.7	0.9	2.4	1.5	0.4	0.4	0.9	1.3	1.1	1.5	0.2
Palpitation	0.2	0.2	0.0	0.6	0.4	0.6	1.9	2.2	2.2	1.7	3.0	1.1	0.9	1.1	2.2	1.7	2.6	0.9
Depression	0.9	0.2	1.1	1.9	1.1	1.3	1.3	1.3	0.9	1.3	1.7	0.9	1.1	1.5	1.9	1.7	2.2	0.9

Dizziness	0.2	0.2	1.1	1.3	0.9	0.6	1.5	1.3	1.3	2.6	2.2	2.4	0.6	1.5	1.3	1.1	1.9	0.9
Nightmare	0.9	1.1	1.9	1.5	2.8	2.4	1.3	1.5	1.1	1.1	1.7	0.6	0.4	1.1	1.9	0.2	0.4	0.6
Memory disturbance	0.2	0.6	0.6	0.6	0.9	1.1	1.5	2.4	1.7	2.4	1.5	0.9	0.6	1.7	1.5	1.5	1.7	0.9
Headache	0.0	0.2	0.6	1.1	1.7	1.1	1.9	1.1	1.3	1.9	1.3	1.7	0.4	1.1	1.3	1.3	1.3	0.9
Frustration	0.4	0.2	0.9	1.9	0.9	1.1	1.3	1.3	0.6	1.3	1.5	0.9	0.4	1.3	1.7	1.5	1.7	0.9
Nasal cavity problem	0.7	1.3	0.9	1.3	1.3	1.3	1.1	1.1	0.7	1.1	1.1	0.4	0.9	1.1	1.1	0.7	1.1	0.7
Abdominal pain	0.3	1.2	1.2	1.2	2.7	1.8	0.9	1.8	1.2	1.2	1.8	1.8	0.9	0.3	2.1	0.9	1.2	0.6
Loss of fun	0.4	0.4	0.2	1.1	1.3	0.2	1.3	0.6	0.4	1.3	1.5	0.2	0.9	1.5	1.9	1.5	0.9	0.6
Ear problem	0.9	1.3	0.4	0.9	2.0	1.8	1.1	1.8	1.1	0.2	1.1	0.7	0.2	0.2	0.2	0.7	1.1	0.0
Muscle pain	0.0	0.0	0.4	0.6	0.9	0.0	1.5	0.6	1.3	1.3	0.4	0.6	0.9	0.6	1.9	0.9	0.9	1.1
Diarrhea	0.6	1.2	0.6	1.2	1.8	0.6	1.2	0.3	0.9	0.6	0.9	1.2	0.6	0.9	1.8	0.9	1.2	0.6
Fever	0.0	1.7	0.9	0.9	1.1	0.0	0.0	0.2	0.9	0.2	0.9	0.4	0.0	0.0	0.6	0.4	0.4	0.0
Brain fog	0.0	0.4	0.0	0.9	0.0	0.4	0.2	0.0	0.6	0.4	0.2	1.1	0.2	0.9	0.9	0.6	1.1	0.2
Skin rash	0.4	1.1	1.1	0.6	0.9	0.4	0.2	0.4	0.2	0.2	1.1	0.0	0.2	0.2	0.2	0.0	0.2	0.2
Joint pain	0.0	0.2	0.2	0.6	0.9	0.6	1.1	0.0	0.4	0.4	0.4	0.2	0.4	0.0	0.9	0.2	0.6	0.2
Nausea	0.0	0.0	0.4	0.9	0.9	0.4	0.9	1.3	0.9	1.3	0.4	0.9	0.4	0.4	1.3	1.3	2.2	0.4
Tinnitus	0.0	0.2	0.0	0.4	0.4	0.0	0.0	0.2	0.4	0.4	0.6	0.2	0.2	0.2	0.4	0.4	0.9	0.0
Voice problem	0.2	0.0	0.4	0.4	0.2	0.0	0.0	0.0	0.2	0.0	0.9	0.7	0.4	0.2	0.2	0.2	0.4	0.0
Skull base pain	0.0	0.2	0.2	0.2	0.2	0.2	0.2	0.4	0.6	0.2	0.6	0.2	0.0	0.2	0.2	0.0	0.0	0.2
Lost taste	0.0	0.2	0.0	0.2	0.0	0.2	0.4	0.4	0.2	0.0	0.0	0.0	0.0	0.2	0.0	0.2	0.4	0.0
Lost smell	0.0	0.2	0.0	0.2	0.0	0.0	0.0	0.0	0.0	0.0	0.0	0.0	0.0	0.4	0.0	0.2	0.4	0.0

Table S3. *P* values distribution of clinical symptoms during the acute and post-COVID-19 phases for 500 children with PASC.

Post \ Acute	Fever	Cough	Rhinorrhea/Nasal congestion	Sore throat	Fatigue	Headache/ Dizziness	Sneezing	Sputum	Chills	Muscle Soreness
Cough	<.0001	<.0001	0.003	0.06	0.09	0.01	<.0001	<.0001	<.0001	<.0001
Fatigue	<.0001	<.0001	0.0004	0.009	0.18	0.02	<.0001	<.0001	<.0001	<.0001
Throat problem	<.0001	<.0001	<.0001	<.0001	0.07	0.32	0.11	0.09	0.01	<.0001
Short of breath	<.0001	<.0001	<.0001	<.0001	0.05	0.24	0.15	0.12	0.02	<.0001
Weakness limiting daily activity	<.0001	<.0001	<.0001	<.0001	0.02	0.16	0.21	0.17	0.01	<.0001
Body weight change	<.0001	<.0001	<.0001	<.0001	0.01	0.10	0.27	0.24	0.02	<.0001
Attention disturbance	<.0001	<.0001	<.0001	<.0001	0.004	0.04	0.66	0.71	0.13	<.0001
Lack of motivation	<.0001	<.0001	<.0001	<.0001	0.0004	0.005	0.94	0.82	0.25	<.0001
Chest pain	<.0001	<.0001	<.0001	<.0001	<.0001	0.001	0.65	0.70	0.53	<.0001
Decreased appetite	<.0001	<.0001	<.0001	<.0001	<.0001	0.0001	0.23	0.30	0.94	0.0003
